# Relationship between different particle size fractions and all-cause and cause-specific emergency ambulance dispatches

**DOI:** 10.1186/s12940-020-00619-5

**Published:** 2020-06-17

**Authors:** Xiaojie Wang, Junzhang Tian, Ziyi Li, Jun Lai, Xin Huang, Yongcong He, Zebing Ye, Guowei Li

**Affiliations:** 1 Center for Clinical Epidemiology and Methodology (CCEM), Guangdong Second Provincial General Hospital, Guangzhou, China; 2 Department of Cardiology, Guangdong Second Provincial General Hospital, Guangzhou, China; 3 Department of Health research methods, Evidence, and Impact (HEI), McMaster University, 1280 Main St West, Hamilton, ON L8S 4L8 Canada

**Keywords:** Particulate matter, Particle size, Emergency ambulance dispatches

## Abstract

**Background:**

Evidence on the relationship between different particle size fractions and emergency ambulance dispatches (EAD) remains limited and sparse.

**Methods:**

We collected daily data of EAD, ambient air pollution and meteorological data from 2014 to 2018 in Guangzhou, China. We used a generalized additive model with covariate adjustments to estimate the associations between different particle size fractions and EAD related to all-cause, cardiovascular diseases, and respiratory diseases. Several subgroup and sensitivity analyses were also performed.

**Results:**

Significant associations were observed between PM_2.5_, PM_2.5–10_, PM_10_ and EADs. A 10 μg/m^3^ increase of PM_2.5,_ PM_2.5–10_, and PM_10_ was associated with an increase of 0.98% (95% CI: 0.67, 1.28%), 2.06% (95% CI: 1.44, 2.68%), and 0.75% (95%CI: 0.53, 0.96%) in all-cause EAD, with an increase of 0.69% (95% CI: 0.00, 1.39%), 2.04% (95% CI: 0.64, 3.45%), and 0.60% (95%CI: 0.11,1.10%) in cardiovascular-related EAD, and an increase of 1.14% (95% CI: 0.25, 2.04%), 2.52% (95% CI: 0.72, 4.35%), and 0.89% (95%CI: 0.25,1.52%) in respiratory-related EAD at lag03, respectively. The results were robust in subgroup and sensitivity analyses.

**Conclusions:**

This study revealed that PM_2.5_, PM_2.5–10_ and PM_10_ were significantly related with risks of all-cause and cause-specific EAD. More evidence of high quality may be needed to further support our results in this ecological study.

## Introduction

Exposures to ambient particulate matter (PM) have been consistently linked with human health [1–3]. The World Health Organization (WHO) reported that ambient PM was a major environmental risk to human health, and it was one of the leading causes of death and disability worldwide, especially in low- and middle-income countries [4].

Most of the previous studies have investigated the health effects of PM pollution with an aerodynamic diameter less than 10 μm (PM_10_) and 2.5 μm (PM_2.5_) [5, 6], and evidence of adverse health effects of these has played an important role in the formulation of air quality standards [7]. Moreover, coarse particulate (PM_2.5–10_), defined as PM between 2.5 and 10 μm in aerodynamic diameter, has been also found to have effects on adverse outcomes [8, 9]. However, even though high levels of particulate air pollutants are often observed, evidence of relationship between PM pollution and human health remains sparse in China, making region-specific results difficult to interpret and compare with those from developed countries.

The majority of prior studies found that PM pollution was associated with mortality, morbidity and hospitalization [10, 11]. While only a limited number of studies focused on the associations between PM pollution and emergency ambulance dispatches (EAD) [12, 13], their findings remained inconsistent. For instance, a study used time-series design in Australia reported that PM_2.5_ and PM_2.5–10_ were significantly and positively associated with EAD [13]. However, another study in the U.S. using a case-crossover design found no association between short-term PM_2.5_ exposure and EAD [14].

Recent epidemiological studies have examined the acute effects of PM pollution on morbidity by using hospital admissions and emergency department visits as indicators. Nevertheless, databases of morbidity information across multiple hospitals for epidemiological studies has not been fully established in China. In recent years, some studies [13, 15] have used EAD as a morbidity indicator to examine the health effects of PM pollution. It is also reported that EAD may be a more appropriate variable to capture the acute effects of air pollution compared with morbidity and mortality [16, 17]. However, the evidence remains sparse and limited about the relationship between different particle size fractions and all-cause and cause-specific EAD in China. Therefore, our purpose was to quantitatively assess the short-term associations between different particle sizes of air pollution and EAD in China. A better understanding of the association between PM pollution and EAD may help inform policy-making of air pollution control and provide better knowledge of the etiology of the related diseases.

## Methods

### Setting

Guangzhou, located in Southern China, belongs to a subtropical humid-monsoon climate. Its annual mean temperature is 22 °C and the average rainfall is 1500–2000 mm. It is the third-largest city in China with 12.7 million people in 2010 [18]. In the last decades, alongside the fast development, serious air pollution has been witnessed among Chinese cities, including Guangzhou [19]. The residents living in urban districts of Guangzhou were selected for this study for two reasons. First, there were nearby air monitoring stations around these districts, and thus, less exposure measurement errors were induced. Second, urban districts have health outcome data of higher quality [20].

### Health data

We obtained the daily counts of EAD due to all-cause, cardiovascular diseases, and respiratory diseases from May 2014 to December 2018 from the Guangzhou Emergency Center. This center serves about seven million people living in urban areas of Guangzhou and is the primary emergency dispatch agency. This center coordinates efforts for approximately 200 ambulances and ensures that emergency responses occur within 30 min after receiving an emergency call, irrespective of the time of day [21]. Ethical approval was not required in this study because no patient privacy was involved and all the data were publicly accessible.

After each emergency call, a standardized data entry form was completed by trained medical personnel. The form included the demographic information, main symptoms, and clinical diagnosis of patients. Diseases were diagnosed by physicians based on the patients’ symptoms, inquiries, and medical examination in standardized procedures where strict quality assurance and quality control were applied. EAD due to traumatic accidents, suicide events, pregnancy and delivery-related events were excluded for analyses in our study. The determination of cardiovascular and respiratory events diagnosis was made by field emergency physicians based on patients’ symptoms and signs. Therefore, it was expected the misclassification rate to be relatively small [22]. Daily counts of EAD were tabulated to construct the time series.

### Air pollution and meteorological data

**Fig. 1:**
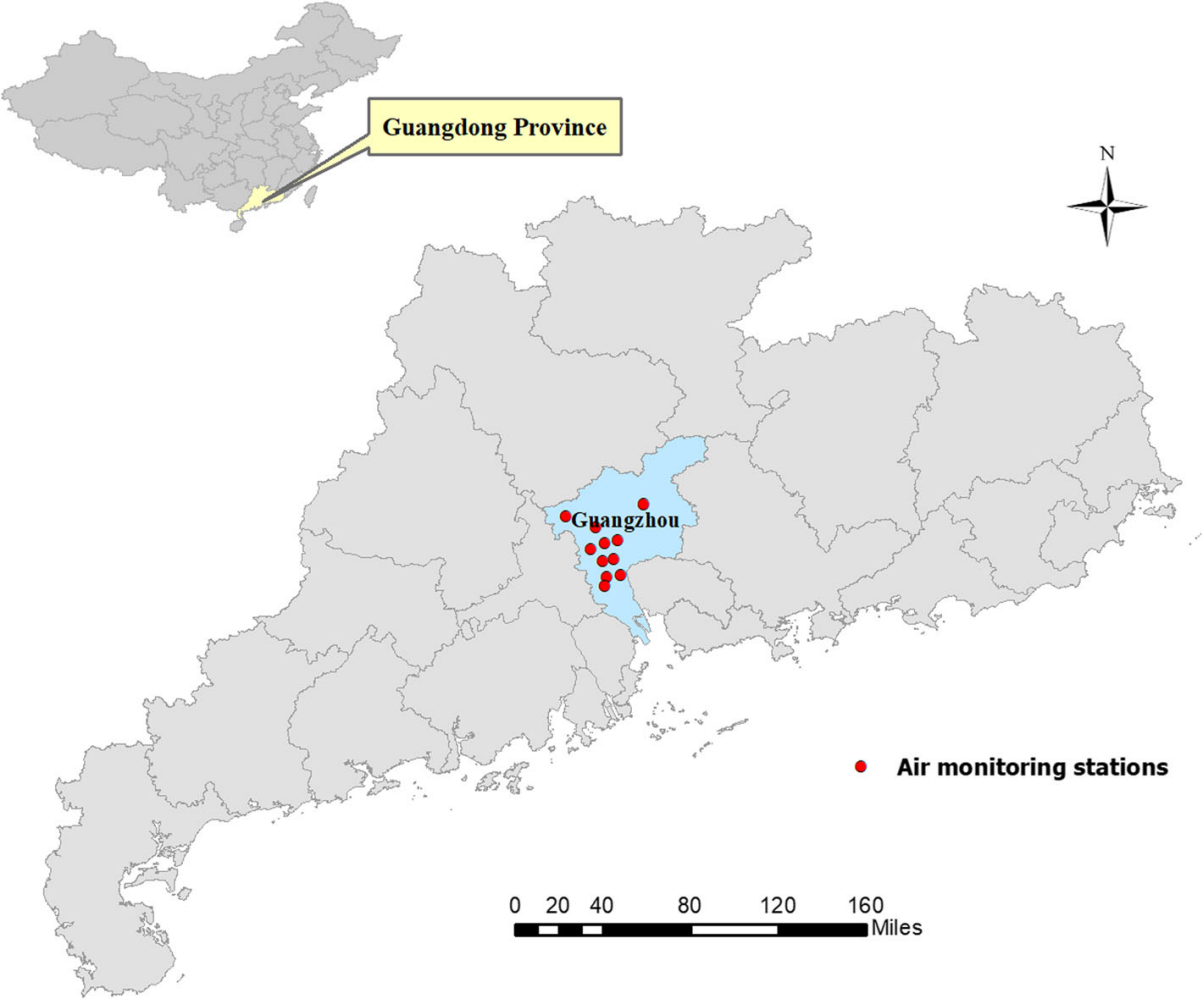
The geographic distribution of the air monitoring stations in Guangzhou, China

Daily concentrations of air pollution were obtained from 11 air monitoring stations in Guangzhou during the period from May 2014 to December 2018 (Their distribution is shown in Fig. 1). We measured the daily mean concentrations of air pollutants based on the data from 24-h monitoring, in which the pollutants included PM_10_, PM_2.5_, nitrogen dioxide (NO_2_), ozone (O_3_) and sulfur dioxide (SO_2_). We estimated PM_2.5–10_ concentrations by subtracting PM_2.5_ from PM_10,_ because PM_10_ was consisted of PM_2.5_ and PM_2.5–10_. Daily mean concentrations of each air pollutant across the stations were used to reflect the general population’s daily exposure. Approximately 1% of observation days had missing data for the particle sizes. We did not perform imputations for missing data in our analyses due to the small percentage of missing data. Air pollution measurement details have been previously described [23].

Daily meteorological data (mean temperature and relative humidity) were obtained from the National Weather Data Sharing System (http://data.cma.cn/).

### Statistical analysis

The EAD data, daily air pollution concentrations and meteorological data were linked by date. We assessed the acute associations between PM pollution and daily counts of EAD due to all-cause, cardiovascular diseases, and respiratory diseases using generalized additive models (GAM). To adjust for the over-dispersion in daily EAD counts, a quasi-Poisson link function was applied. This analysis strategy has been widely used previously [24, 25]. Day of the week (DOW) and public holidays (PH) were controlled for as categorical variables. Smoothing spline functions with different degrees of freedom were used to control for all the nonlinear time-dependent variables including temporal trends and meteorological factors. Similar to previous studies, we selected our model specification a priori for the degrees of freedom (df) for long-term and seasonal trends and other meteorological variables [26]. Specifically, we applied 8 df per year for temporal trends, 6 df for temperatures of the current day and the previous three days moving averages (Temp03), and 3 df for current day’s relative humidity [27].

We first generated a base model without PM pollutants. The base model was written as below:

$$ \log \left[\mathrm{E}\left(\mathrm{Yt}\right)\right]=\upalpha +\mathrm{s}\left(\mathrm{t}, df=6/\mathrm{year}+\mathrm{s}\right(\mathrm{Temp}03, df=6+\kern0.5em \mathrm{s}\left(\mathrm{Humidity}, df=3\right)+{\upbeta}_1\ast \mathrm{DOW}+{\upbeta}_2\ast \mathrm{PH} $$

where E(Yt) was the expected EAD count on day t, α was the model intercept, s() indicated the smoother, t represented the time in order to adjust for long-term and seasonal trends, and β was the regression coefficient.

We then included the concentrations of the three size fractions in the regression models separately to examine their associations. Due to the high correlation between the particulate pollutants, we fitted three separate regression models.

To examine the lagged effects of air pollution, models with different lag structures were constructed beginning with the same day (lag0) up to five days lag before (lag5) and with multi-day lags [moving average of current day and previous 1, 3, and 5 days (lag01, lag03, and lag05)]. A maximum of five days was considered because there was little evidence of associations beyond five days in China [28]. We also considered multi-lag days, and the effects at each lag day were finally reported for each size fraction [29].

We conducted stratified analyses to investigate the possible effect modification of the PM pollution-EAD associations by sex (males vs. females), age strata (< 65 years vs. ≥65), and season (cold vs. warm). Warm season was defined as April to September, and cold season was October to March. We tested whether the subgroup differences between effect estimates of the strata were significant by calculating the 95% confidence interval (CI) as shown below:

$$ \left({Q}_1-{Q}_2\right)\pm 1.96\sqrt{{\left(\mathrm{S}{\mathrm{E}}_1\right)}^2+{\left(\mathrm{S}{\mathrm{E}}_2\right)}^2} $$

where Q_1_ and Q_2_ represented the estimates for the two categories (e.g., males and females), and SE_1_ and SE_2_ are their corresponding standard errors [30].

To support the robustness of our results, we conducted a series of sensitivity studies. The main findings obtained from the current study were assessed by varying the degrees of freedom in the smooth functions for temporal trends and further by adjusting for gaseous air pollutants (NO_2,_ O_3,_ and SO_2_). We conducted another sensitivity analysis by controlling longer lag days’ temperature and relative humidity (up to 21 days), because previous studies had found that the temperature may have a longer lag of health effect [19, 31].

We conducted all the analyses using the “mgcv” package in R (version 3.6.0; R Development Core Team, Vienna, Austria). The effect estimates were reported as excess risk (ER) in daily EAD associated with per 10 μm/m^3^ increase in each PM pollutant, where ER was defined as [relative risk (RR)-1]*100%. Statistical significance was defined as *p* < 0.05.

## Results

A total of 586,197 EAD were recorded during the study period, 56,827 of which was for cardiovascular diseases and 38,829 for respiratory diseases. Table 1 summarizes the distribution of daily EAD counts, air pollution, and meteorological variables. On average, there were 391 EAD per day due to all-cause diseases, 38 EAD due to cardiovascular diseases, and 26 EAD due to respiratory diseases. In brief, the daily concentrations of PM_2.5_, PM_2.5–10_, PM_10_, NO_2_, O_3_, and SO_2_ were 36.0, 20.5, 56.5, 44.1, 45.2, and 15.9 μg/m^3^ during 2014–2018, respectively. The daily mean temperature was 22.2 °C and relative humidity was 79.6%.

**Table 1:** Description of air pollutants, meteorological factors and emergency ambulance dispatches between 2014 to 2018 in Guangzhou

	No. of missing days	Daily mean (SD)	Quantiles				
			Min	P25	P50	P75	Max
Pollutants, μg/m^3^							
PM_2.5_	17	36.0 (19.0)	4.6	22.0	31.7	46.8	148.3
PM_2.5–10_	17	20.5 (9.2)	1.3	14.5	18.7	25.0	62.2
PM_10_	17	56.5 (26.1)	10.0	37.5	50.4	71.1	208.3
NO_2_	17	44.1 (17.4)	11.7	31.7	40.4	52.1	155.0
O_3_	17	45.2 (24.9)	3.5	26.7	40.6	59.6	139.2
SO_2_	17	15.9 (13.3)	2.8	9.2	12.1	16.4	85.4
Meteorological factors							
Temperature (°C)	0	22.2 (6.1)	3.4	17.5	23.8	27.4	32.8
Relative Humidity (%)	0	79.6 (10.2)	31.0	74.0	81.0	87.0	100.0
Emergency ambulance dispatches, n							
All-cause	0	391 (38.9)	267	364	388	413	553
Cardiovascular diseases	0	38 (8.0)	19	32	37	43	68
Respiratory diseases	0	26 (7.3)	9	21	25	30	53

The spearman’s correlation coefficients between the air pollutants are shown in Table S1. Generally, PM_2.5_ was strongly correlated with PM_2.5–10_ and PM_10_ (r = 0.71 with PM_2.5–10_, r = 0.97 with PM_10_). The correlations of particulate matter with O_3_ and SO_2_ were relatively low with an r ranging from 0.09 to 0.33, while the correlations of NO_2_ with particulate matter were high (r ranging from 0.69 to 0.78).

**Fig. 2:**
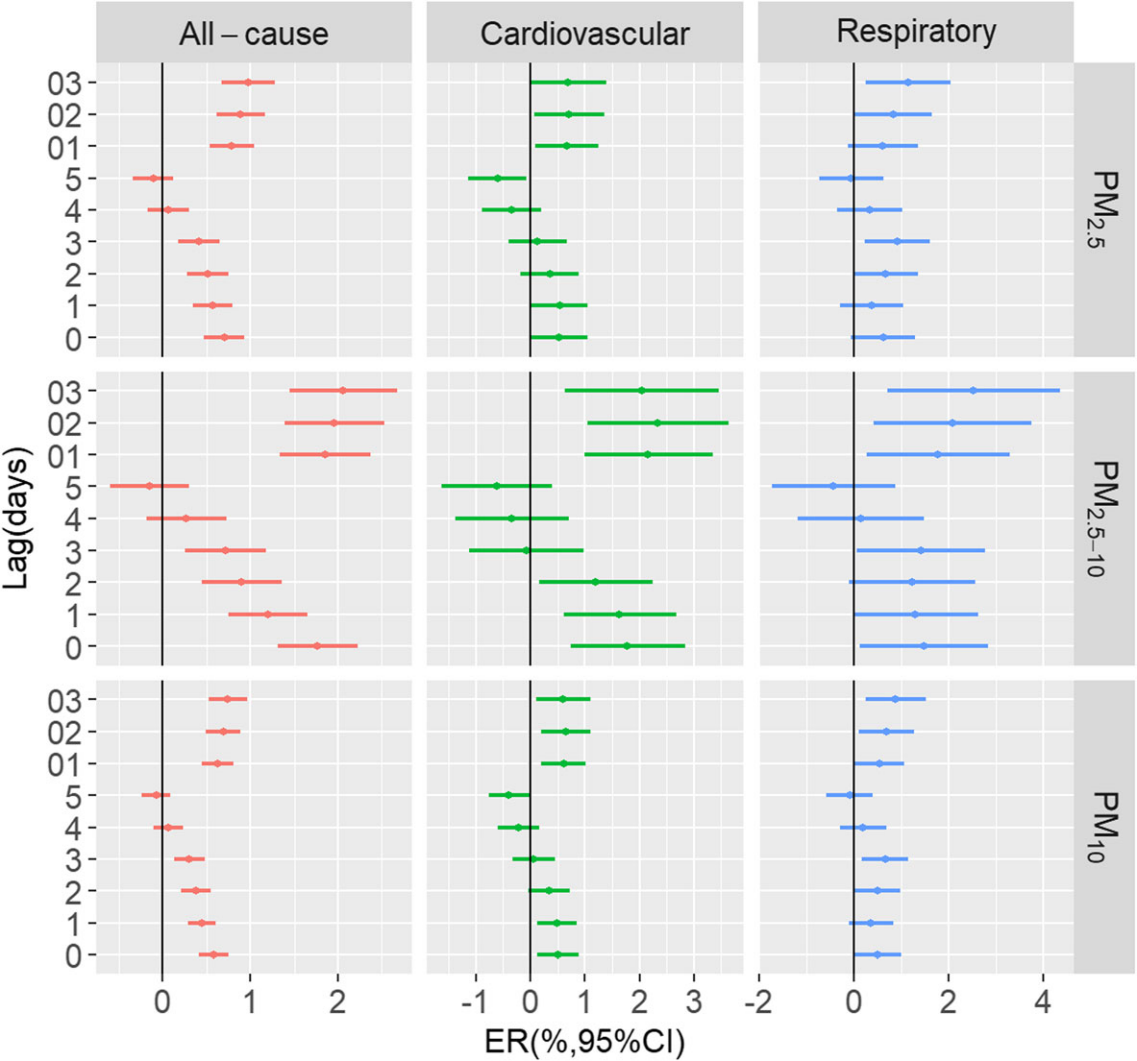
Excess risk of emergency ambulance dispatches related to all-cause, cardiovascular diseases, and respiratory diseases for per 10 μg/m^3^ increment in particulate pollutants with different lag days (single lags for the current day (lag0) to 5 days before the current day (lag5) and multiday lags for the current day and prior 1 day before (lag01), 2 days (lag02) or 3 days (lag03))

Figure 2 presents the association between PM pollution with different size fractions and EAD over different lag days in the single-pollutant regression models. In general, we found the largest effects at lag03; therefore, in the subsequent analyses, we mainly reported the effects of lag03. We observed a similar lag-patterns for EAD of all-cause, cardiovascular diseases, and respiratory diseases. In the single-day lag patterns, the effects of different size fractions on EAD decreased from lag0 to lag5 days, and negative associations were observed from lag3 to lag5 days in all-cause and cardiovascular diseases. In cumulative lag structures, PM_2.5_, PM_2.5–10_, and PM_10_ were significantly associated with EAD for all-cause, cardiovascular diseases and respiratory diseases. For example, the ER for a 10 μg/m^3^ increase in daily PM_2.5,_ PM_2.5–10_ and PM_10_ at lag01 for cardiovascular diseases were 0.66% (95% CI: 0.09, 1.24%), 2.16% (0.99, 3.34%), and 0.61% (95% CI: 0.20, 1.02%), respectively.

Table 2 illustrates the ER of EAD associated with per 10 μg/m^3^ increase in different size fractions in single and two-pollutant models at lag03. In single pollutant models, we observed statistically significant associations of different particle sizes with EAD for all-cause, cardiovascular diseases and respiratory diseases. In two-pollutant models, the results remained robust after adjusting for NO_2_, O_3_, and SO_2_, albeit the effects were slightly reduced in some models. Meanwhile, the associations between different particle sizes and cardiovascular diseases weakened and became nonsignificant after controlling for NO_2_. Moreover, the effect of PM_2.5_ on cardiovascular diseases became non-significant when adjusted for SO_2_.

**Table 2:** Excess risk and 95% confidence intervals of emergency ambulance dispatches for each 10 μg/m^3^ increment in PM pollution at lag03 in single and two-pollutant models in Guangzhou

Pollutants	Models	All-cause	Cardiovascular	Respiratory
PM_2.5_				
	Single pollutant model	0.98 (0.67, 1.28)	0.69 (0.00, 1.39)	1.14 (0.25, 2.04)
	Two-pollutant models			
	Control for NO_2_	0.46 (0.09, 0.83)	0.07 (− 0.78, 0.92)	1.21 (0.12, 2.31)
	Control for O_3_	1.11 (0.79, 1.44)	0.74 (0.00, 1.49)	1.35 (0.40, 2.31)
	Control for SO_2_	0.96 (0.65, 1.27)	0.61 (− 0.10, 1.33)	1.03 (0.12, 1.94)
PM_2.5–10_				
	Single pollutant model	2.06 (1.44, 2.68)	2.04 (0.64, 3.45)	2.52 (0.72, 4.35)
	Two-pollutant models			
	Control for NO_2_	1.05 (0.30, 1.79)	1.07 (−0.64, 2.81)	2.82 (0.60, 5.10)
	Control for O_3_	2.26 (1.61, 2.91)	2.17 (0.70, 3.66)	2.89 (0.99, 4.82)
	Control for SO_2_	2.03 (1.40, 2.66)	1.89 (0.47, 3.34)	2.29 (0.45, 4.15)
PM_10_				
	Single pollutant model	0.75 (0.53, 0.96)	0.60 (0.11, 1.10)	0.89 (0.25, 1.52)
	Two-pollutant models			
	Control for NO_2_	0.38 (0.12, 0.65)	0.18 (−0.44, 0.80)	1.02 (0.22, 1.83)
	Control for O_3_	0.85 (0.62, 1.08)	0.65 (0.13, 1.18)	1.05 (0.38, 1.73)
	Control for SO_2_	0.74 (0.52, 0.96)	0.55 (0.04, 1.05)	0.80 (0.16, 1.45)

Table 3 shows the relationships between PM pollution and EAD stratified by gender, age and season. We found that the estimates varied by aforementioned factors, but the difference was only statistically significant for different age groups and seasons in the effects of PM_2.5–10_ on EAD. The estimates of PM with different sizes on all-cause EAD were higher in males than females, while the effects reversed in cardiovascular diseases and respiratory diseases. In different age groups, EAD for all-cause, cardiovascular diseases, and respiratory diseases showed higher effects in the older group (age ≥ 65). In season groups, PM_2.5_-EAD associations were stronger in the cold season; however, PM_2.5–10_-EAD and PM_10_-EAD associations were stronger in the warm season.

**Table 3:** Excess risks and 95% confidence intervals of emergency ambulance dispatches associated with each 10 μg/m^3^ increase in PM_2.5_, PM_2.5–10_, and PM_10_ at lag03, stratified by gender, age and season^*^

Pollutants	Stratum	All-cause	Cardiovascular	Respiratory
PM_2.5_				
	Gender			
	Male	1.01 (0.56, 1.46)	0.38 (−0.78, 1.55)	0.80 (−0.67, 2.29)
	Female	0.54 (0.08, 0.99)	0.48 (−0.49, 1.45)	1.03 (−0.14, 2.22)
	Age			
	< 65	0.63 (0.14, 1.13)	0.37 (−0.71, 1.47)	−0.67 (−2.18, 0.86)
	≥ 65	1.06 (0.61, 1.51)	0.99 (0.02, 1.97)	1.81 (0.73, 2.89)
	Season			
	Warm	0.84 (0.31, 1.37)	0.74 (−0.62, 2.12)	1.93 (0.25, 3.64)
	Cold	1.02 (0.65, 1.39)	1.06 (0.17, 1.95)	2.05 (0.93, 3.17)
PM_2.5–10_				
	Sex			
	Male	1.79 (0.88, 2.71)	1.24 (−0.68, 3.20)	1.93 (− 0.43, 4.34)
	Female	1.45 (0.54, 2.38)	2.15 (−0.18, 4.53)	2.72 (−0.24, 5.76)
	Age			
	< 65	**0.54 (−0.45, 1.54)**	2.03 (0.10, 4.01)	**−0.42 (−3.42, 2.66)**
	≥ 65	**2.62 (1.72, 3.54)**	2.32 (0.14, 4.55)	**3.52 (1.34, 5.74)**
	Season			
	Warm	2.77 (1.41, 4.15)	**5.60 (2.08, 9.23)**	4.36 (2.15, 6.61)
	Cold	1.84 (1.11, 2.59)	**1.58 (−0.15, 3.33)**	3.82 (− 0.51, 8.34)
PM_10_				
	Sex			
	Male	0.73 (0.41, 1.05)	0.40 (−0.29, 1.08)	0.74 (−0.30, 1.79)
	Female	0.45 (0.13, 0.77)	0.46 (−0.36, 1.28)	0.76 (−0.07, 1.59)
	Age			
	< 65	0.38 (0.03, 0.74)	0.48 (−0.29, 1.25)	−0.39 (−1.46, 0.69)
	≥ 65	0.86 (0.54, 1.18)	0.75 (0.06, 1.44)	1.34 (0.58, 2.10)
	Season^a^			
	Warm	0.76 (0.35, 1.17)	0.95 (−0.10, 2.01)	1.53 (0.76, 2.31)
	Cold	0.72 (0.46, 0.98)	0.71 (0.10, 1.32)	1.50 (0.20, 2.82)

^*^Data in bold indicated that the differences between the effect estimates of the strata were statistically significant (*p* < 0.05)

^a^ Warm season was defined as April to September, and cold season was October to March

Sensitivity analyses (Table S2) indicated that the results were insensitive when the df were changed. For example, regarding the effects of PM_2.5_, when the df of “temporal trends” was replaced from 6 to 8, the corresponding ERs were 0.89% (95% CI: 0.58, 1.20%) for all-cause EAD, 0.74% (95% CI: 0.02, 1.46%) for cardiovascular-related EAD, and 1.47% (95% CI: 0.55, 2.39%) for respiratory-related EAD at lag03. In another sensitivity analysis adjusting for temperature of longer lag days (up to 21 days), the effects remained largely consistent. Similar results were also observed when we controlled for relative humidity of longer lag days.

## Discussion

Although previous evidence has linked PM pollution with adverse health outcomes worldwide [32, 33], only a few have investigated the PM effects on EAD. To our knowledge, our study is the first attempt to simultaneously examine the effects of particle size on the risk of EAD for all-cause, cardiovascular diseases, and respiratory diseases in China.

Ambient PM pollution is a critical public health concern in China. Our study observed that short-term exposures to PM_2.5_, PM_2.5–10_, and PM_10_ were significantly associated with risks of all EAD related to all-cause, cardiovascular, and respiratory diseases, which shared some similarities with previous studies [16, 34]. For example, one study in Japan reported that short-term exposure to PM_2.5_ was associated with EAD for all-cause (OR = 1.008, 95% CI: 1.002, 1.014), and respiratory diseases (OR = 1.027, 95% CI: 1.007, 1.048) at lag0 [34]. Besides, Xia’s study found that PM_2.5_ and PM_2.5–10_ had effects on EAD due to cardiovascular diseases, with an OR of 1.07 (95% CI: 1.04, 1.10) and 1.05 (95% CI: 1.03, 1.07) at lag1, respectively [35]. Previous studies had reported several underlying mechanisms that could explain the increased EAD associated with PM pollution. For instance, PM had been found to be associated with increased systemic inflammatory responses, plasma viscosity [36], changes in blood pressure [37], decreased heart rate variability and increased cardiac arrhythmias [38].

Our study found that the effects of PM_2.5–10_ were higher than PM_2.5_ on all outcomes, which was consistent with results reported in previous studies. For instance, a time-series study in Japan [39] found that each 10 μg/m^3^ increase in PM_2.5–10_ was associated with all-cause mortality (OR of 1.016, 95%CI: 1.011, 1.022), which was higher than in PM_2.5_ (OR of 1.006, 95%CI: 1.003, 1.009)_._ Furthermore, while most research and regulatory agencies have directed their attention to PM_2.5_, our findings also emphasized the importance of monitoring and assessing PM_2.5–10_.

In our study, the effects of PM pollution on EAD seemed not to be confounded by O_3_ and SO_2_. However, the relationship between PM pollution and cardiovascular diseases became nonsignificant after adjusting for NO_2_. Because of moderate to high correlations between air pollutants (Table S1), it was difficult to ascertain their potential effects especially given the potential multicollinearity issue. In two pollutant models involving PM_2.5_ and NO_2_, this problem was even more apparent [40].

We estimated a larger effect of PM_2.5_, PM_2.5–10_ and PM_10_ on EAD due to all-cause, cardiovascular diseases, and respiratory diseases among older population than younger. Consistent results on the age-specific effects of particulate air pollution were reported previously [41, 42]. Part of the possible underlying explanations included that the older individuals usually had pre-existing illnesses and were in bad health status, and thus had diminished ability to respond to the acute exposure to high level of air pollution [43].

Our gender-stratified results illuminated that at the same concentration levels of air pollution, a higher risk of EAD for all-cause was observed among males compared with females, which was consistent with recent studies [35, 44]. However, females had a slightly higher risk of cause-special EAD (such as cardiovascular diseases and respiratory diseases) than males, although the differences were not statistically significant. The discrepancy might be due to biological and behavioral differences between males and females, such as physiopathological responses to air pollution and time spent outdoors/indoor [45].

Furthermore, regarding the season-stratified analysis, the effects of PM_2.5–10_ on EAD were significantly stronger in the warm season. The potential reasons remained largely unclear. However, several factors may account for aggravated effects in the warm seasons. For example, high temperatures could increase blood viscosity and cholesterol, contributing to thrombosis [46]; people preferred to stay outdoors in warm seasons than in cold seasons, which increased the risk of exposure to PM [47]; and thermoregulation system responded heat stress by increasing sweating, minute ventilation, and cardiac output, all of which tend to increase the uptake and distribution of air pollutants in the human body [15, 48], to mention a few.

Several limitations of this study should be noted. We used air pollution concentration measured at fixed air monitoring stations to represent personal exposure, making exposure misclassification possible; however, using daily air pollutant concentrations (averaged over all available stations) as measures of exposure was considered acceptable in ecological studies [49]. Furthermore, as an ecological study, this study was limited in controlling unmeasured potential confounders because of the limited data. For instance, some important variables including living environment, history of diseases, and lifestyle patterns were not available in this study. It was unknown about whether these variables would represent a threat to validity of our results because no analyses could be performed. Moreover, our study was performed in just one Chinese city due to data accessibility, which may impair the generalizability of our results. Even though it had been reported that EAD could serve as a morbidity indicator and even be more appropriate to capture the short-term effects of air pollutants than mortality, the associations between EAD and morbidity and mortality required more justifications. Unfortunately no analyses could be conducted in our study due to lack of the information on mortality and morbidity. Further evidence would be largely needed to assess the relationship between the use of EAD and morbidity and mortality outcomes.

## Conclusion

In conclusion, PM_2.5_, PM_2.5–10_ and PM_10_ were found to be significantly related with EAD, while gender, age, and season might be important effect modifiers in these relationships. More evidence of high quality may be needed to further support our results in this ecological study.

## Supplementary information

**Additional file 1: Table S1**. Pearson correlation coefficients between the daily mean concentrations of air pollutants between 2014 to 2018 in Guangzhou. **Table S2**. Sensitivity analysis for emergency ambulance dispatches associated with each 10 μg/m^3^ increment of different size fractions of particulate matter pollution at lag03.

## Data Availability

Please contact author for data requests.

## Abbreviations

EAD: Emergency ambulance dispatches
CI: Confidential intervals
RR: Relative risk
ER: Excess risk
WHO: World Health Organization
PM: Particulate matter
PM_10_: Particulate matter with an aerodynamic diameter ≤ 10 mm
PM_2.5–10_: Particulate matter between 2.5 and 10 μm in aerodynamic diameter
PM_2.5_: Particulate matter with an aerodynamic diameter ≤ 2.5 mm SO_2_: Sulfur dioxide
NO_2_: Nitrogen dioxide
O_3_: Ozone
GAM: Generalized additive models
DOW: Day of the week
PH: Public holidays
df: Degrees of freedom
