# Supplementary material for: Relationship between different particle size fractions and all-cause and cause-specific emergency ambulance dispatches
Source: Environ Health. 2020 Jun 17;19:69. doi: 10.1186/s12940-020-00619-5 (PMC7301562; doi:10.1186/s12940-020-00619-5)
Supplement: Supplementary file 1 — Additional file 1: Table S1. Pearson correlation coefficients between the daily mean concentrations of air pollutants between 2014 to 2018 in Guangzhou. Table S2. Sensitivity analysis for emergency ambulance dispatches associated with each 10 μg/m3 increment of different size fractions of particulate matter pollution at lag03. [file 12940_2020_619_MOESM1_ESM.docx]

Table S1. Pearson correlation coefficients between the daily mean concentrations of air pollutants between 2014 to 2018 in Guangzhou.

| Pollutants | PM_2.5_ | PM_2.5-10_ | PM_10_ | NO_2_ | O_3_ | SO_2_ |
| --- | --- | --- | --- | --- | --- | --- |
| PM_2.5_ | 1.00 | - | - | - | - | - |
| PM_2.5-10_ | 0.71^*^ | 1.00 | - | - | - | - |
| PM_10_ | 0.97^*^ | 0.84^*^ | 1.00 | - | - | - |
| NO_2_ | 0.74^*^ | 0.69^*^ | 0.78^*^ | 1.00 | - | - |
| O_3_ | 0.29^*^ | 0.33^*^ | 0.33^*^ | 0.02 | 1.00 | - |
| SO_2_ | 0.09^*^ | 0.10^*^ | 0.10^*^ | 0.03^*^ | -0.01^*^ | 1.00 |

^*^P<0.05

Table S2. Sensitivity analysis for emergency ambulance dispatches associated with each 10μg/m^3^ increment of different size fractions of particulate matter pollution at lag03.

| Pollutants | Models | All-cause | Cardiovascular | Respiratory |
| --- | --- | --- | --- | --- |
| PM_2.5_ |  |  |  |  |
|  | df=5 for temporal trends | 1.07 (0.75, 1.38) | 0.79 (0.09, 1.50) | 1.38 (0.48, 2.28) |
|  | df=7 for temporal trends | 0.96 (0.66, 1.26) | 0.79 (0.09, 1.49) | 1.24 (0.35, 2.14) |
|  | df=8 for temporal trends | 0.89 (0.58, 1.20) | 0.74 (0.02, 1.46) | 1.47 (0.55, 2.39) |
|  | df=5 for temperature | 0.95 (0.65, 1.26) | 0.69 (-0.01, 1.39) | 1.08 (0.18, 1.98) |
|  | df=7 for temperature | 0.96 (0.66, 1.26) | 0.69 (-0.01, 1.39) | 1.11 (0.22, 2.01) |
|  | df=8 for temperature | 0.96 (0.66, 1.26) | 0.69 (-0.01, 1.39) | 1.10 (0.21, 2.00) |
|  | Lag021 temperature | 0.73 (0.36, 1.10) | 0.12 (-0.91, 0.68) | 0.58 (-0.39, 1.56) |
|  | Lag021 relative humidity | 1.46 (1.11, 1.80) | 0.99 (0.19, 1.80) | 1.57 (0.57, 2.59) |
| PM_2.5-10_ |  |  |  |  |
|  | df=5 for temporal trends | 2.54 (1.93, 3.16) | 2.59 (1.22, 3.99) | 2.87 (1.10, 4.68) |
|  | df=7 for temporal trends | 2.20 (1.59, 2.82) | 2.05 (0.64, 3.48) | 2.78 (0.97, 4.63) |
|  | df=8 for temporal trends | 2.18 (1.54, 2.82) | 2.12 (0.66, 3.60) | 3.67 (1.78, 5.58) |
|  | df=5 for temperature | 2.01 (1.40, 2.63) | 2.02 (0.62, 3.43) | 2.44 (0.64, 4.28) |
|  | df=7 for temperature | 2.00 (1.39, 2.62) | 2.03 (0.64, 3.45) | 2.38 (0.58, 4.21) |
|  | df=8 for temperature | 2.01 (1.40, 2.62) | 2.04 (0.64, 3.45) | 2.41 (0.62, 4.24) |
|  | Lag021 temperature | 2.06 (1.35, 2.78) | 0.91 (-0.61, 2.47) | 2.28 (0.40, 4.19) |
|  | Lag021 relative humidity | 2.86 (2.18, 3.55) | 2.15 (0.60, 3.72) | 3.08 (1.14, 5.06) |
| PM_10_ |  |  |  |  |
|  | df=5 for temporal trends | 0.86 (0.64, 1.08) | 0.73 (0.24, 1.22) | 1.06 (0.42, 1.69) |
|  | df=7 for temporal trends | 0.74 (0.53, 0.95) | 0.64 (0.15, 1.14) | 0.96 (0.33, 1.59) |
|  | df=8 for temporal trends | 0.72 (0.50, 0.94) | 0.63 (0.12, 1.14) | 1.19 (0.54, 1.84) |
|  | df=5 for temperature | 0.73 (0.51, 0.94) | 0.60 (0.10, 1.09) | 0.85 (0.21, 1.48) |
|  | df=7 for temperature | 0.73 (0.52, 0.94) | 0.60 (0.11, 1.09) | 0.85 (0.22, 1.49) |
|  | df=8 for temperature | 0.73 (0.52, 0.94) | 0.60 (0.11, 1.09) | 0.85 (0.22, 1.49) |
|  | Lag021 temperature | 0.64 (0.38, 0.90) | 0.07 (-0.50, 0.63) | 0.60 (-0.09, 1.29) |
|  | Lag021 relative humidity | 1.12 (0.87, 1.36) | 0.79 (0.22, 1.36) | 1.21 (0.50, 1.92) |
